# Phylogenetic and morphological classification of *Ophiocordyceps* species on termites from Thailand

**DOI:** 10.3897/mycokeys.56.37636

**Published:** 2019-07-29

**Authors:** Kanoksri Tasanathai, Wasana Noisripoom, Thanyarat Chaitika, Artit Khonsanit, Sasitorn Hasin, Jennifer Luangsa-ard

**Affiliations:** 1 Plant Microbe Interaction Research Team, Bioscience and Biotechnology for Agriculture, BIOTEC, NSTDA, 113 Thailand Science Park, Phahonyothin Rd., Khlong Nueng, Khlong Luang, Pathum Thani 12120, Thailand Bioscience and Biotechnology for Agriculture Pathum Thani Thailand; 2 Innovation of Environmental Management, College of Innovative Management, Valaya Alongkorn Rajabhat University under the Royal Patronage, Khlong Luang, Pathum Thani 12120, Thailand Valaya Alongkorn Rajabhat University under the Royal Patronage Pathum Thani Thailand

**Keywords:** Entomopathogenic fungi, Hypocreales, Isoptera, Ophiocordycipitaceae, Taxonomy

## Abstract

Seven new species occurring on termites are added to *Ophiocordyceps* – *O. asiatica*, *O. brunneirubra*, *O. khokpasiensis*, *O. mosingtoensis*, *O. pseudocommunis*, *O. pseudorhizoidea* and *O. termiticola*, based on morphological and molecular phylogenetic evidence. *O. brunneirubra* possesses orange to reddish-brown immersed perithecia on cylindrical to clavate stromata. *O. khokpasiensis*, *O. mosingtoensis* and *O. termiticola* have pseudo-immersed perithecia while *O. asiatica*, *O. pseudocommunis* and *O. pseudorhizoidea* all possess superficial perithecia, reminiscent of *O. communis* and *O. rhizoidea*. Phylogenetic analyses based on a combined dataset comprising the internal transcribed spacer regions (ITS) and the largest subunit (LSU) of the ribosomal DNA, partial regions of the elongation factor 1-α (*TEF*) and the largest and second largest subunits for the RNA polymerase genes (*RPB1*, *RPB2*) strongly support the placement of these seven new species in *Ophiocordyceps*.

## Introduction

The entomopathogenic genus *Ophiocordyceps* was established by Petch in 1931. His description was based on four specimens including *O. blattae* Petch, the type species, occurring on a cockroach collected from Sri Lanka, *O. unilateralis* (Tul. & C. Tul.) Petch on ants, *O. peltata* (Wakef.) Petch on Coleoptera larva (*Cryptorhynchus* sp.) and *O. rhizoidea* (Höhn.) Petch on Coleoptera larva. The distinction of the genus from *Cordyceps* Fr. was made due the presence of clavate asci that gradually narrowed to a thickened apex, as opposed to the cylindrical asci in many *Cordyceps* species. The ascospores in *Ophiocordycepssensu* Petch are elongated fusoid, multi-septate that remain whole after discharge. Sung et al. (2007) emended the definition of *Ophiocordyceps* to contain the anamorphic genera *Hirsutella* Pat., *Hymenostilbe* Petch, *Paraisaria* Samson & Brady and *Syngliocladium* Petch, with the stromata or subiculum of the teleomorphs mostly darkly pigmented [e.g. *O. acicularis* (Ravenel) Petch, *O. heteropoda* (Kobayasi) G.H. Sung, J.M. Sung, Hywel-Jones & Spatafora, *O. entomorrhiza* (Dicks.) G.H. Sung, J.M. Sung, Hywel-Jones & Spatafora, *O. unilateralis* species complex] and sometimes brightly coloured [e.g. *O. irangiensis* (Moureau) G.H. Sung, J.M. Sung, Hywel-Jones & Spatafora, *O. nutans* (Pat.) G.H. Sung, J.M. Sung, Hywel-Jones & Spatafora, *O. sphecocephala* (Klotzsch ex Berk.) G.H. Sung, J.M. Sung, Hywel-Jones & Spatafora]. The ascospores are usually cylindrical, multi-septate that could either dissociate into part-spores (*O. sphecocephala*, *O. nutans*) or remain whole ascospores (*O. unilateralis*). To date, *Ophiocordyceps* is the most speciose genus in Ophiocordycipitaceae with 235 names of accepted species (Spatafora et al. 2015; Khonsanit et al. 2018; Luangsa-ard et al. 2018). Most Asian species of *Ophiocordyceps* have fibrous, hard and pliant to wiry, dark coloured stromata with superficial to immersed perithecia (Kobayasi 1941; Kobmoo et al. 2012, 2015; Luangsa-ard et al. 2018).

Only a few species of entomopathogenic fungi have been reported from termites. Currently accepted species include *Ophiocordyceps bispora* (Stifler) G.H. Sung, J.M. Sung, Hywel-Jones & Spatafora on *Macrotermes* from Tanzania, *O. koningsbergeri* (Penz. & Sacc.) G.H. Sung, J.M. Sung, Hywel-Jones & Spatafora, known only from the type locality (Java, Indonesia) (Kobayasi 1941), *C. termitophila* Kobayasi & Shimizu known from Japan and Taiwan (Kobayasi and Shimizu 1978) and *O. octospora* (M. Blackw. & Gilb.) G.H. Sung, J.M. Sung, Hywel-Jones & Spatafora on *Tenuirostritermes* from Mexico (Blackwell and Gilbertson 1981). Penzig and Saccardo (1904) found *O. koningsbergeri* to be similar to *O. myrmecophila* (Ces., in Rabenshorst 1858) G.H. Sung, J.M. Sung, Hywel-Jones & Spatafora in that it had a terminal, globose head with immersed perithecia.

Termites (Isoptera) are one of the eusocial and soil insects that have successfully evolved since the Cretaceous Period and are classified into 7 families, 14 subfamilies, 280 genera and 2,500 species (Pearce 1999). They occur throughout tropic and subtropic regions and can also be found in many temperate areas and semi-arid environments of the world (Eggleton et al. 2000). Termites are abundant in Thailand and are found in natural forests as well as urban areas, mostly considered as serious pests of wooden constructions. Current records of termite species from Thailand have been 199 species, 39 genera, 10 subfamilies and 4 families (Sornnuwat et al. 2004). Relationships between termites and fungi are classified into two categories. Firstly, termites cultivate fungi (*Termitomyces* spp.) in their fungus gardens within the subterranean nest or mound of fungus-growing termites (subfamily Macrotermitinae). Secondly, a parasitic interaction, in which fungi infect and consume termites as food for its nutrient value (Abe et al. 2000). Some species of fungi are known as pathogens of termites and they can be used as potential agents of biological control for each of the host’s (i.e. termites) specificities (Rath 2000).

In surveys of entomopathogenic fungi in national parks and community forests collections of termite pathogens, most with superficial perithecia and rarely with immersed perithecia were found. The phenotypic characters of the collections in having wiry and pliant, darkly pigmented stromata identifies them primarily to be members of the Ophiocordycipitaceae, mostly as *Ophiocordyceps communis*. The aims of this study are (1) to clarify the relationships of these collections to known members of the Ophiocordycipitaceae, (2) to uncover hidden species in *O. communis* species complex and (3) to describe new taxa to accommodate species diversity in *Ophiocordyceps*.

## Material and methods

### Collection and isolation

Species occurring on termites (Isoptera) were found in the ground. The specimens were excavated carefully so as not to lose the host, which could be buried as deep as 15 cm under the ground and were placed in small plastics boxes before returning to the laboratory for isolation. The materials were examined under a stereomicroscope (OLYMPUS SZ61, Olympus Corporation, Japan). The fertile heads of the specimens containing mature perithecia were carefully placed over the Potato Dextrose Agar plate (PDA; fresh diced potato 200 g, dextrose 20 g, agar 15 g, in 1 litre distilled water). These were placed in a plastic box with moist tissue paper overnight to create a humid chamber. The following morning plates were examined with a stereomicroscope to check the discharged ascospores. Discharged ascospores were examined daily for germination and also for fungal contaminants.

### Morphological study

The newly collected specimens were noted and photographed in the field using a digital Nikon D5100 camera and were taken to the laboratory and photographed using an Olympus SZX12 before they were placed in a moist chamber to facilitate ascospore discharge. The colour of the freshly collected specimens and cultures were characterised with the colour standard of the Online Auction Colour Chart. One to two perithecia were removed from the stroma and mounted on a glass slide using lactophenol cotton blue to measure their sizes and shapes, as well as the sizes and shapes of the asci and ascospores. Cultures on PDA, Potato Sucrose Agar plate (PSA: potato 200 g/l, sucrose 20 g/l, calcium carbonate 5g/l, agar 20g/l) and quarter strength Sabouraud Dextrose Yeast Agar (SDYA/4; Difco) were observed using light microscopy (Olympus SZ60, CX 30) daily to check for germination and contamination for 2–3 wks. Colony growth rates and characteristics (colour, texture, pigmentation) under dark/light condition (L:D = 14:10) were recorded and photos were taken using the Nikon D5100 camera.

For micro-morphological description, microscope slide cultures were prepared from a block of media (PDA, PSA and SDYA/4, ca. 5 × 5 mm^2^) inoculated with the fungus and overlaid by a glass coverslip. The cultures were incubated at 25 °C. Observations, measurements of the conidiogenous cells and conidia of the asexual morphs and photographs were taken with an Olympus DP11 microscope.

### Host identification

Dead termite hosts were identified, based on morphological characteristics, such as mandibulate mouthparts, antennae, shape of head and thoraxes. The identification of dead insects was conducted after pure cultures were acquired. Termites were identified by using the extant families of Isoptera after Sornnuwat et al. (2004) and Krishna et al. (2013).

### DNA extraction, PCR amplification and sequencing

Cultivation of fungi for molecular work. – Pure cultures were grown on PDA. After approximately 2 wks, the plates were checked for contaminants and small agar blocks were inoculated into sterile Erlenmeyer flasks containing 50 ml Sabouraud Dextrose Broth (Difco) and incubated for 1–2 wks at 25 °C without shaking. Mycelium was then harvested by filtration and washed several times with sterile distilled water. Filtered mycelium was lyophilised. The material was extracted from mycelium by a modified CTAB method as previously described (Luangsa-ard et al. 2004, 2005).

PCR amplification. – Five nuclear loci including the nuc rDNA region encompassing the internal transcribed spacers 1 and 2, along with the 5.8S rDNA (ITS), nuc 28S rDNA (*LSU*), the translation elongation factor 1-α gene (*TEF*) and the genes for RNA polymerase II largest (*RPB1*) and second largest (*RPB2*) subunits were sequenced. PCR primers used to amplify the gene regions for this study were: ITS5, ITS4 for ITS, LROR and LR7 for *LSU* (White et al. 1990), 983F and 2218R for *TEF*, CRPB1 and RPB1Cr for *RPB*1, RPB2-5F2 and RPB2-7Cr for *RPB*2 (Castlebury et al. 2004). The PCR reaction mixture consisted of 1× PCR buffer, 200 μM of each of the four dNTPs, 2.5 mM MgCl_2_, 0.4 M Betaine, 1 U Taq DNA Polymerase, recombinant (Thermo Scientific, US) and 0.2 μM of each primer in a total volume of 50 μl. PCR cycle conditions were as previously described in Sung et al. (2007). PCR amplicons were visualised by ethidium bromide staining after gel electrophoresis of 4 µl of the product in 0.8% agarose gel. Quantification of the PCR products was performed using a standard DNA marker of known size and weight. PCR products were purified using Qiagen columns (QIAquick PCR Purification Kit). Purified PCR products were sequenced with the PCR amplification primers.

### Sequencing alignment and phylogenetic analyses

The DNA sequences, generated in this study, were examined for ambiguous bases using BioEdit 7.2.5 (Hall 2004) and then submitted to GenBank (Table 1). The dataset of taxa in Cordycipitaceae was assembled from previously published studies (Sung et al. 2007; Kepler et al. 2017) and were downloaded from GenBank for the construction of the phylogenetic tree (Table 1). Alignments were performed using MUSCLE 3.6 software with default settings (Edgar 2004). Sequences of *Cordyceps kyusyuensis* and *Cordyceps militaris* in the *Cordycipitaceae* were used as the outgroup.

Maximum Likelihood (ML) analyses was performed with RAxML-HPC2 on XSEDE v8.2.10 (Stamatakis 2014) with the use of GAMMA Model parameters. The reliability of ML internal branches was assessed using a non-parametric bootstrap method with 1000 replicates. Bayesian (BI) phylogenetic inference was performed with MrBayes on XSEDE v3.2.6 (Ronquist and Huelsenbeck 2003) using the GTR+I+G model as selected by MrModeltest v2.2 (Nylander 2004). The chain length of the Bayesian analyses was 5,000,000 generations, sampled every 1000 generations and a burn-in of 10% of the total run. Maximum parsimony analysis was conducted on the combined dataset using PAUP 4.0b10 (Swofford 2002).

**Table 1.** List of species and GenBank accession numbers of sequences used in this study.

Species	Strain nr.	Host/Substratum	GenBank accession no.				
			ITS rDNA	LSU	*TEF*	*RPB1*	*RPB2*
* Cordyceps kyusyuensis *	EFCC 5886	Lepidoptera	–	EF468813 ^1^	EF468754 ^1^	EF468863 ^1^	EF468917 ^1^
* Cordyceps militaris *	OSC 93623	Lepidoptera	JN049825 ^1^	AY184966 ^1^	DQ522332 ^1^	DQ522377 ^1^	AY545732 ^1^
* Drechmeria gunnii *	OSC 76404	Lepidoptera (pupa)	–	AF339522 ^2^	AY489616 ^2^	AY489650 ^2^	DQ522426 ^2^
* Drechmeria sinensis *	CBS 567.95	Nematoda	AJ292417 ^2^	AF339545 ^2^	DQ522343 ^2^	DQ522389 ^2^	DQ522443 ^2^
Hirsutella cf. haptospora	ARSEF 2228	Diptera: Itonididae	KM652166 ^3^	KM652118 ^3^	KM652001 ^3^	KM652041 ^3^	–
* Hirsutella citriformis *	ARSEF 1446	Hemiptera; Cixiidae	KM652154 ^3^	KM652106 ^3^	KM651990 ^3^	KM652031 ^3^	–
	ARSEF 1035	Hemiptera; Cixiidae	KM652153 ^3^	KM652105 ^3^	KM651989 ^3^	KM652030 ^3^	–
* Hirsutella cryptosclerotium *	ARSEF 4517	Hemiptera; Pseudococcidae	KM652157 ^3^	KM652109 ^3^	KM651992 ^3^	KM652032 ^3^	–
* Hirsutella fusiformis *	ARSEF 5474	Coleoptera: Curculionidae	–	KM652110 ^3^	KM651993 ^3^	KM652033 ^3^	–
* Hirsutella gigantea *	ARSEF 30	Hymenoptera: Pamphiliidae	–	–	JX566980 ^3^	KM652034 ^3^	–
* Hirsutella haptospora *	ARSEF 2226	Acari: Uropodina	KM652159 ^3^	–	KM651995 ^3^	KM652036 ^3^	–
* Hirsutella illustris *	ARSEF 5539	Hemiptera: Aphididae	KM652160 ^3^	KM652112 ^3^	KM651996 ^3^	KM652037 ^3^	–
* Hirsutella lecaniicola *	ARSEF 8888	Hemiptera: Coccidae	KM652162 ^3^	KM652114 ^3^	KM651998 ^3^	KM652038 ^3^	–
* Hirsutella liboensis *	ARSEF 9603	Lepidoptera: Cossidae	KM652163 ^3^	KM652115 ^3^	–	–	–
* Hirsutella necatrix *	ARSEF 5549	Acari	KM652164 ^3^	KM652116 ^3^	KM651999 ^3^	KM652039 ^3^	–
* Hirsutella nodulosa *	ARSEF 5473	Lepidoptera; Pyralidae	KM652165 ^3^	KM652117 ^3^	KM652000 ^3^	KM652040 ^3^	–
* Hirsutella radiata *	ARSEF 1369	Diptera	–	KM652119 ^3^	KM652002 ^3^	KM652042 ^3^	–
*Hirsutella repens* nom. inval.	ARSEF 2348	Hemiptera: Delphacidae	KM652167 ^3^	KM652120 ^3^	KM652003 ^3^	–	–
* Hirsutella rhossiliensis *	ARSEF 2931	Tylenchida: Heteroderidae	KM652168 ^3^	KM652121 ^3^	KM652004 ^3^	KM652043 ^3^	–
* Hirsutella satumaensis *	ARSEF 996	Lepidoptera: Pyralidae	KM652172 ^3^	KM652125 ^3^	KM652008 ^3^	KM652047 ^3^	–
*Hirsutella* sp.	ARSEF 8378	Hemiptera: Cixiidae	–	KM652127 ^3^	KM652010 ^3^	KM652049 ^3^	–
* Hirsutella strigosa *	ARSEF 2197	Hemiptera: Cicadellidae	KM652175 ^3^	KM652129 ^3^	KM652012 ^3^	KM652050 ^3^	–
	ARSEF 2044	Hemiptera: Delphacidae	KM652174 ^3^	KM652128 ^3^	KM652011 ^3^	–	–
* Hirsutella subulata *	ARSEF 2227	Lepidoptera: Microlepidoptea	KM652176 ^3^	KM652130 ^3^	KM652013 ^3^	KM652051 ^3^	–
* Hirsutella thompsonii *	ARSEF 257	Acari; Eriophyidae	KM652182 ^3^	KM652136 ^3^	KM652019 ^3^	KM652054 ^3^	–
	ARSEF 414	Acari; Eriophyidae	KM652184 ^3^	KM652139 ^3^	KM652021 ^3^	KM652056 ^3^	–
	ARSEF 3323	Acari: Tenuipalpidae	KM652188 ^3^	KM652143 ^3^	KM652024 ^3^	KM652059 ^3^	–
	ARSEF 3482		KM652189 ^3^	KM652144 ^3^	KM652025 ^3^	KM652060 ^3^	–
	ARSEF 253	Acari: Eriophyidae	KM652179 ^3^	KM652133 ^3^	KM652016 ^3^	–	–
	ARSEF 256	Acari: Eriophyidae	KM652181 ^3^	KM652135 ^3^	KM652018 ^3^	KM652053 ^3^	–
	ARSEF 258	Acari: Eriophyidae	–	KM652137 ^3^	KM652020 ^3^	KM652055 ^3^	–
	ARSEF 2800	Acari	KM652187 ^3^	KM652142 ^3^	KM652023 ^3^	KM652058 ^3^	–
Hirsutella thompsonii “var. synnematosa”	ARSEF 1947	Acari: Tarsonemidae	KM652191 ^3^	KM652146 ^3^	KM652026 ^3^	–	–
	ARSEF 5412	Acari: Tetranychidae	KM652193 ^3^	KM652148 ^3^	–	–	–
Hirsutella thompsonii var. vinacea	ARSEF 254	Acari: Eriophyidae	KM652194 ^3^	KM652149 ^3^	KM652028 ^3^	KM652062 ^3^	–
* Hirsutella versicolor *	ARSEF 1037	Hemiptera: Membracidae	–	KM652150 ^3^	KM652029 ^3^	KM652063 ^3^	–
* Ophiocordyceps acicularis *	OSC 110988	Coleoptera (larva)	–	EF468804 ^2^	EF468745 ^2^	EF468853 ^2^	–
	OSC 110987	Coleoptera (larva)	–	EF468805 ^2^	EF468744 ^2^	EF468852 ^2^	–
* Ophiocordyceps agriotidis *	ARSEF 5692	Coleoptera (larva)	JN049819 ^2^	DQ518754 ^2^	DQ522322 ^2^	DQ522368 ^2^	DQ522418 ^2^
* Ophiocordyceps aphodii *	ARSEF 5498	Coleoptera	–	DQ518755 ^2^	DQ522323 ^2^	–	DQ522419 ^2^
* Ophiocordyceps appendiculata *	NBRC 106960	Coleoptera (larva)	JN943326 ^2^	JN941413 ^2^	–	JN992462 ^2^	–
*** Ophiocordyceps asiatica ***	**BCC 30516**	**Termitidae (adult termite)**	**MH754722**	**MH753675**	**MK284263**	**MK214105**	**MK214091**
	**BCC 86435**	**Termitidae (adult termite)**	**MH754723**	**MH753676**	–	**MK214106**	**MK214092**
*** Ophiocordyceps communis ***	**BCC 1842**	**Termitidae (adult termite)**	**MH754726**	**MH753680**	**MK284266**	**MK214110**	**MK214096**
	**BCC 1874**	**Termitidae (adult termite)**	**MH754725**	**MH753679**	**MK284267**	**MK214109**	**MK214095**
	**BCC 2754**	**Termitidae (adult termite)**	**MH754727**	**MH753681**	**MK284268**	**MK214111**	**MK214097**
* Ophiocordyceps brunneipunctata *	OSC 128576	Coleoptera (Elateridae larva)	–	DQ518756 ^2^	DQ522324 ^2^	DQ522369 ^2^	DQ522420 ^2^
*** Ophiocordyceps brunneirubra ***	**BCC 14384**	**Termitidae (adult termite)**	**MH754736**	**MH753690**	**GU797121**	**MK751465**	**MK751468**
	**BCC 14478**	**Termitidae (adult termite)**	**MH754734**	**MH753688**	**GU797122**	**MK751466**	**MK214102**
	**BCC 14477**	**Termitidae (adult termite)**	**MH754735**	**MH753689**	**GU797123**	**MK751467**	**MK214103**
* Ophiocordyceps dipterigena *	OSC 151911	Diptera (adult fly)	–	KJ878886 ^4^	KJ878966 ^4^	KJ879000 ^4^	–
* Ophiocordyceps elongata *	OSC 110989	Lepidoptera (larva)	–	EF468808 ^2^	EF468748 ^2^	EF468856 ^2^	–
* Ophiocordyceps gracilioides *	HUA 186095	Coleoptera (Elateridae larva)	–	–	KM411994 ^2^	KP212914 ^2^	–
	HUA 186092	Coleoptera (Elateridae larva)	–	KJ130992 ^2^	–	KP212915 ^2^	–
* Ophiocordyceps gracilis *	EFCC 8572	Lepidoptera (larva)	JN049851 ^2^	EF468811 ^2^	EF468751 ^2^	EF468859 ^2^	EF468912 ^2^
	EFCC 3101	Lepidoptera (larva)	–	EF468810 ^2^	EF468750 ^2^	EF468858 ^2^	EF468913 ^2^
* Ophiocordyceps granospora *	BCC 82255	Hymenoptera (*Polyrhachis* sp.)	MH028143 ^4^	MH028156 ^4^	MH028183 ^4^	MH02816 ^4^	MH02817 ^4^
* Ophiocordyceps heteropoda *	EFCC 10125	Hemiptera (cicada nymph)	JN049852 ^2^	EF468812 ^2^	EF468752 ^2^	EF468860 ^2^	EF468914 ^2^
* Ophiocordyceps irangiensis *	BCC 82793	Hymenoptera (*Polyrhachis illaudata*)	MH028141 ^4^	–	MH028185 ^4^	MH028163 ^4^	MH02817 ^4^
	BCC 82795	Hymenoptera (*Polyrhachis* sp.)	MH028142 ^4^	–	MH028186 ^4^	MH028164 ^4^	MH02817 ^4^
* Ophiocordyceps khaoyaiensis *	BCC 82796	Hymenoptera (*Polyrhachis armata*)	MH028150 ^4^	MH028153 ^4^	MH028187 ^4^	MH028165 ^4^	MH02817 ^4^
	BCC 82797	Hymenoptera (*Polyrhachis armata*)	MH028151 ^4^	MH028154 ^4^	MH028188 ^4^	MH028166 ^4^	MH02817 ^4^
*** Ophiocordyceps khokpasiensis ***	**BCC 48071**	**Termitidae (adult termite)**	**MH754728**	**MH753682**	**MK284269**	**MK214112**	–
	**BCC 48072**	**Termitidae (adult termite)**	**MH754729**	**MH753683**	**MK284270**	**MK214113**	–
	**BCC 1764**	**Termitidae (adult termite)**	**MH754730**	**MH753684**	**MK284271**	**MK214114**	**MK214098**
* Ophiocordyceps konnoana *	EFCC 7315	Coleoptera (larva)	–	–	EF468753 ^2^	EF468861 ^2^	EF468916 ^2^
* Ophiocordyceps longissima *	NBRC 108989	Hemiptera (cicada nymph)	AB968407 ^1^	AB968421 ^1^	AB968585 ^1^	–	–
	EFCC 6814	Hemiptera (cicada nymph)	–	EF468817 ^2^	EF468757 ^2^	EF468865 ^2^	–
*** Ophiocordyceps mosingtoensis ***	**BCC 30904**	**Termitidae (adult termite)**	**MH754732**	**MH753686**	**MK284273**	**MK214115**	**MK214100**
	**BCC 36921**	**Termitidae (adult termite)**	**MH754731**	**MH753685**	**MK284272**	**MK214116**	**MK214099**
* Ophiocordyceps myrmecophila *	CEM **1710**	Hymenoptera (Adult ant)	–	KJ878894 ^4^	KJ878974 ^4^	KJ879008 ^4^	–
* Ophiocordyceps myrmicarum *	ARSEF 11864	Hymenoptera: Formicidae	–	–	JX566973 ^3^	KJ680151 ^3^	–
* Ophiocordyceps nigrella *	EFCC 9247	Lepidoptera (larva)	JN049853 ^2^	EF468818 ^2^	EF468758 ^2^	EF468866 ^2^	EF468920 ^2^
*** Ophiocordyceps pseudocommunis ***	**BCC 16757**	**Termitidae (adult termite)**	**MH754733**	**MH753687**	**MK284274**	**MK214117**	**MK214101**
* Ophiocordyceps pseudocommunis *	NHJ 12581	Termitidae (adult termite)	–	EF468831 ^3^	EF468775 ^3^	–	EF468930 ^3^
	NHJ 12582	Termitidae (adult termite)	–	EF468830 ^3^	EF468771 ^3^	–	EF468926 ^3^
*** Ophiocordyceps pseudorhizoidea ***	**BCC 48879**	**Termitidae (adult termite)**	**MH754720**	**MH753673**	**MK284261**	**MK214104**	**MK214089**
	**BCC 86431**	**Termitidae (adult termite)**	**MH754721**	**MH753674**	**MK284262**	**MK751469**	**MK214090**
	**NHJ 12522**	**Termitidae (adult termite)**	**JN0498572**	**EF4688252**	**EF4687642**	**EF4688732**	**EF4689232**
	**NHJ 12529**	**Termitidae (adult termite)**	–	**EF4688242**	**EF4687652**	**EF4688722**	**EF4689222**
* Ophiocordyceps pulvinata *	TNS-F-30044	Hymenoptera	–	–	GU904209 ^5^	GU904210 ^5^	–
* Ophiocordyceps ravenelii *	OSC 110995	Coleoptera (larva)	–	DQ518764 ^2^	DQ522334 ^2^	DQ522379 ^2^	–
* Ophiocordyceps robertsii *	KEW 27083	Lepidoptera (Hepialidae larva)	–	EF468826 ^2^	EF468766 ^2^	–	–
* Ophiocordyceps satoi *	J7	Hymenoptera (*Polyrhachis lamellidens*)	–	KX713599 ^5^	KX713683 ^5^	KX713711 ^5^	–
	J19	Hymenoptera (*Polyrhachis lamellidens*)	–	KX713601 ^5^	KX713684 ^5^	KX713710 ^5^	–
* Ophiocordyceps sinensis *	ARSEF 6282	Lepidoptera; Hepialidae	KM652173 ^3^	KM652126 ^3^	KM652009 ^3^	KM652048 ^3^	–
	EFCC 7287	Lepidoptera; Hepialidae (larva)	JN049854 ^2^	EF468827 ^2^	EF468767 ^2^	EF468874 ^2^	EF468925 ^2^
* Ophiocordyceps sobolifera *	KEW 78842	Hemiptera (cicada nymph)	JN049855 ^2^	EF468828 ^2^	–	EF468875 ^2^	DQ522432 ^2^
* Ophiocordyceps spataforae *	NHJ 12525	Hemiptera	–	EF469078 ^6^	EF469063 ^6^	EF469092 ^6^	EF469111 ^6^
	OSC 128575	Hemiptera	–	EF469079 ^6^	EF469064 ^6^	EF469093 ^6^	EF469110 ^6^
* Ophiocordyceps sphecoceplala *	NBRC 101416	Hymenoptera (adult wasp)	–	JN941443 ^4^	–	JN992432 ^4^	–
* Ophiocordyceps stylophora *	OSC 111000	Coleoptera; Elateridae (larva)	JN049828 ^2^	DQ518766 ^2^	DQ522337 ^2^	DQ522382 ^2^	–
*** Ophiocordyceps termiticola ***	**BCC 1920**	**Termitidae (adult termite)**	**MH754724**	**MH753678**	**MK284265**	**MK214108**	**MK214094**
	**BCC 1770**	**Termitidae (adult termite)**	**GU723780**	**MH753677**	**MK284264**	**MK214107**	**MK214093**
* Ophiocordyceps unilateralis *	OSC 128574	Hymenoptera	–	DQ518768 ^2^	DQ522339 ^2^	DQ522385 ^2^	DQ522436 ^2^
* Ophiocordyceps xuefengensis *	GZUHHN 13	Lepidoptera; Phassus nodus (larva)	KC631804 ^2^	–	KC631790 ^2^	KC631795 ^2^	–
* Ophiocordyceps yakusimensis *	HMAS 199604	Hemiptera; (cicada nymph)	–	KJ878902 ^2^	–	KJ879018 ^2^	KJ878953 ^2^
* Purpureocillium lilacinum *	CBS 284.36	Soil	AY624189 ^2^	–	EF468792 ^2^	EF468898 ^2^	EF468941 ^2^
	CBS 431.87	Nematoda	AY624188 ^2^	EF468844 ^2^	EF468791 ^2^	EF468897 ^2^	EF468940 ^2^

*Note.* The accession numbers marked in bold font refer to sequences new in this study or have been generated by our group in Thailand. ^1^Ban et al. (2015), ^2^Sanjuan et al. (2015), ^3^Simmons et al. (2015), ^4^Khonsanit et al. (2018), ^5^Araújo et al. (2018), ^6^Luangsa-ard et al. (2018)

## Results

### Phylogenetic analysis

We obtained 96 new sequences from 20 specimens (Table 1). The combined dataset of five genes consisted of 4013 bp (ITS 527 bp, *LSU* 824 bp, *TEF* 901 bp, *RPB1* 874 bp, *RPB2* 854 bp) and 99 taxa were analysed.

**Figure 1.**
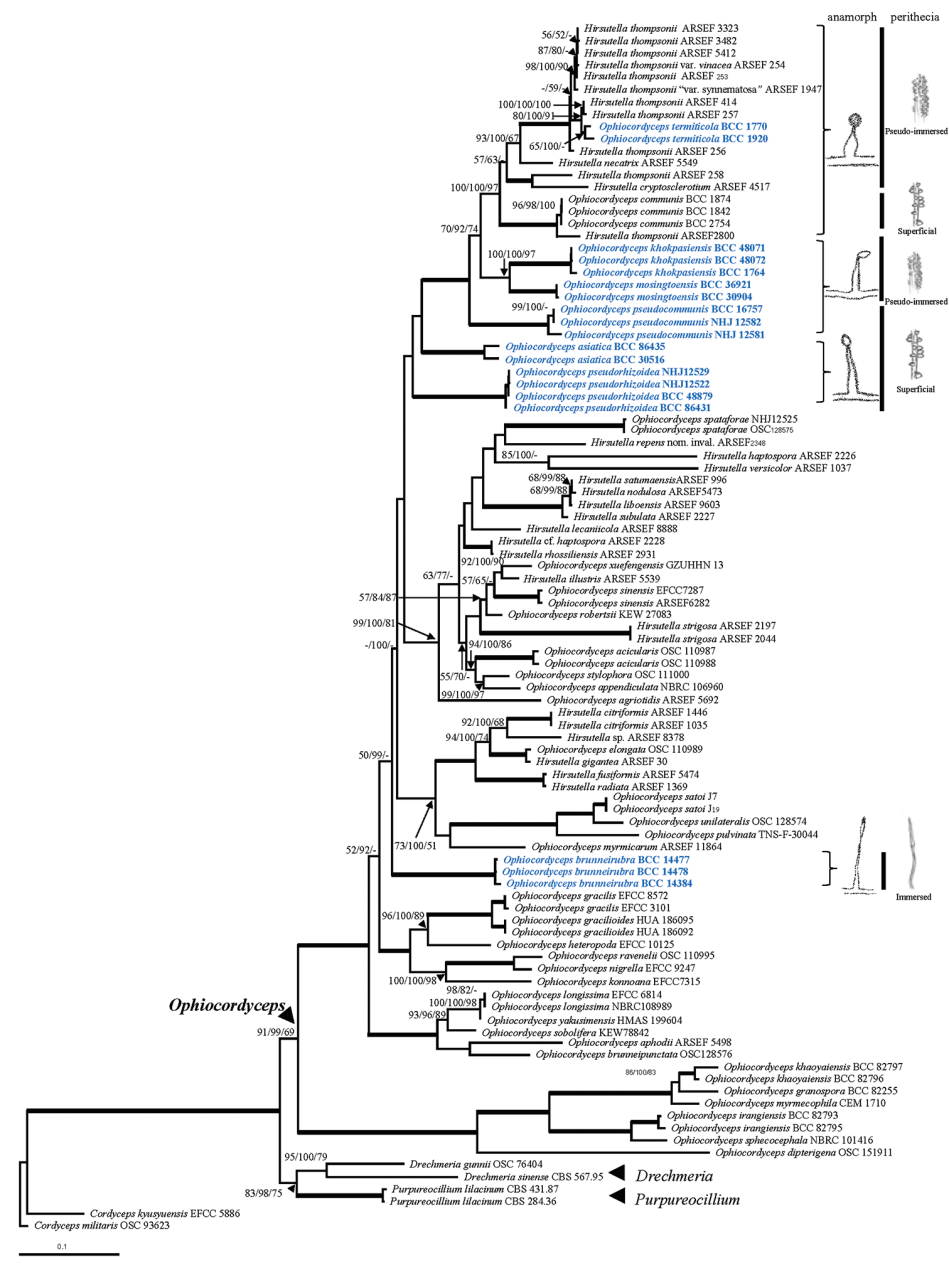
Phylogenetic tree based on combined data set of ITS, LSU, *TEF*, *RPB1* and *RPB2* sequences showing the relationship of seven new species on termites from Thailand with other species of *Ophiocordyceps*. Numbers above lines at significant nodes represent Maximum Likelihood bootstrap values, Bayesian posterior probabilities and MP bootstrap values. Bold lines mean support for the tree analyses were 100%.

The ML and BI analyses displayed similar topologies resolving seven new species in *Ophiocordyceps* (Fig. 1). The final ML optimisation likelihood = -51972.210615 and tree length = 5.567057. The parameters included base frequencies—A = 0.227576, C = 0.299408, G = 0.284488, T = 0.188528 and the rate matrix for the substitution model: [AC] = 1.240734, [A-G] = 2.882814, [A-T] = 0.983408, [C-G] = 1.338444, [C-T] = 5.445401, [G-T] = 1. 000000. In the BI analyses, the model selected was GTR+I+G, -lnL = 52578.1641. The parameters used included base frequencies—freqA = 0.1918, freqC = 0.3427, freqG = 0.2769, freqT = 0.1886 and the rate matrix for the substitution model: [AC] = 1.2356, [A-G] = 3.1814, [A-T] = 1.1029, [C-G] = 1.1220, [C-T] = 4.7720, [G-T] = 1.0000. The MP analyses resulted in 32 equally most parsimonious trees with 4013 characters, 1912 of which are constant, 355 are variable and parsimony-uninformative, while 1746 are parsimony-informative and tree length has 10669 steps (CI, 0.348; RI, 0.689; RC, 0.240; HI, 0.652).

## Taxonomy

### Ophiocordyceps asiatica

Taxon classificationFungiHypocrealesOphiocordycipitaceae

Tasanathai, Noisripoom & Luangsa-ard sp. nov.

cace421c-40ed-5b6e-a477-576bc3a7b5d5

MycoBank MB 831297

Figure 2

#### Typification.

THAILAND. Nakhon Ratchasima Province, Khao Yai National Park; 14°711'N, 101°421'E; on termite; 21 May 2008; K. Tasanathai, S. Mongkolsamrit, B. Thongnuch, P. Srikitikulchai, R. Ridkaew, A. Khonsanit (holotype BBH 38718 dried culture; ex-type living culture, BCC 30516). GenBank: ITS = MH754722, LSU = MH753675, *TEF* = MK284263, *RPB1* = MK214105, *RPB2* = MK214091

**Figure 2.**
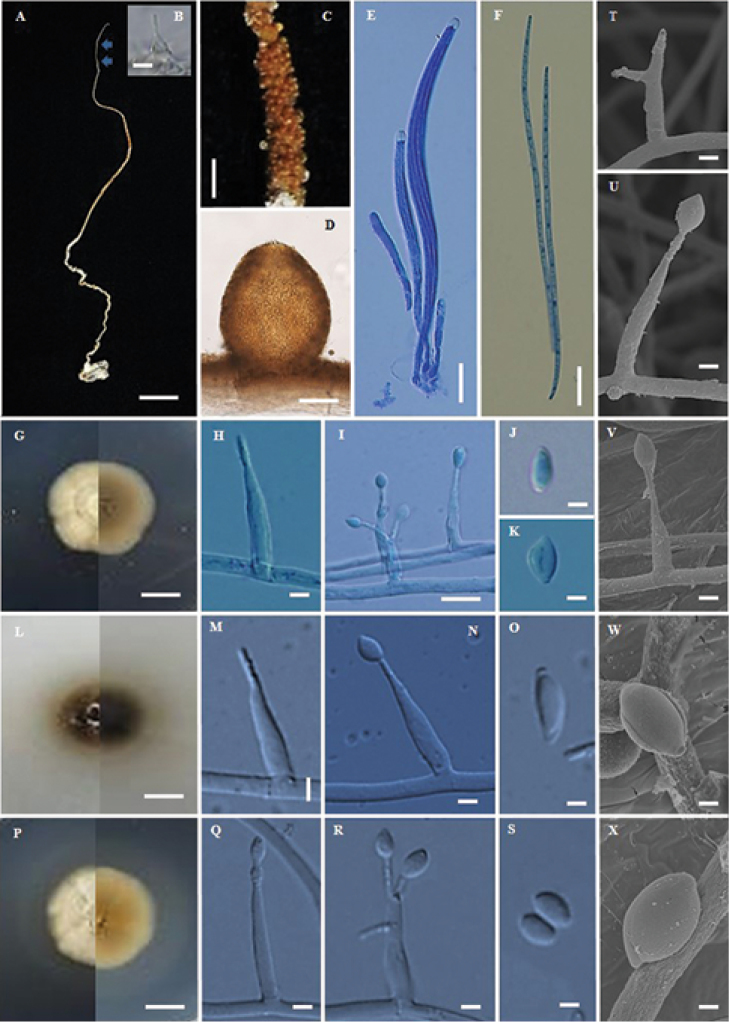
*Ophiocordyceps asiatica* (BBH38718, BCC30516) **A** stroma of fungus emerging from termite **B** phialide on specimen **C** part of stroma showing perithecia **D** perithecium **E** asci **F** ascospores **G** colony on PDA at 20 d obverse and reverse **H, I** phialides with conidia on PDA**J, K** conidium **L** colony on PSA at 20 d obverse and reverse **M, N** phialides with conidia on PSA**O** conidium **P** colony on SDYA/4 at 20 d obverse and reverse **Q, R** phialides with conidia **S** conidia **T–X** scanning electron micrographs of phialides with conidia on PDA. Scale bars: 10 mm (**A, G, L, P**); 5 μm (**B**); 1 mm (**C**); 8 μm (**D**); 15 μm (**E**); 10 μm (**F, I**); 3 μm (**H, J, K, M, N, S, T, V**); 2 μm (**O, Q, R, U, W, X**).

#### Etymology.

‘*asiatica*’ referring to Asia.

#### Description.

Stroma solitary, simple, filiform, up to 15 cm long, 1 mm wide, orange-brown (oac48-50), ca. 10 cm emerging above leaf litter, 5 cm buried in the soil. Asexual state (*Hirsutella*) produced at the terminal part of the stroma, ca. 2 cm long, light brown to grey. *Perithecia* superficial covering middle part of stroma, globose to subglobose, (240–)261.5–302(–320) × (180–)205–240.5(–260) µm. *Asci* 8-spored, filiform, (92.5–)104–143.5(–175) × 5–6.5 µm with cap, 2 × 2 µm. *Ascospores* whole, filiform, (80–)100–122.5(–132.5) × 1–2 µm, with septate. Asexual state *Hirsutella*, phialides arising singly or laterally from the hyphae along the terminal part of the stroma, (9–)9.5–13(–15) × (3–)3.5–4.8(–5) µm, *conidia* hyaline, fusiform, 4–5×2–3 µm.

#### Culture characteristics.

Colonies on PDA, attaining a diam. of 27 mm after 20 d at 25 °C, mycelium sparse to abundant, grey in the middle to pale brown. *Conidiogenous cells* developing directly on the aerial mycelium, swollen towards the base, hyaline, smooth, tapering gradually towards the apex, which often forms a thin warty neck (1 µm), monophialidic or rarely polyphialidic 15–18.5(–20) × 2–3 µm µm. *Conidia* aseptate, hyaline, smooth, arising from phialides at the apex of each neck, fusiform, (7–)7.6–9 × 2–3 µm, with a mucous sheath.

Colonies on PSA, attaining a diam. of 25 mm after 20 d at 25 °C, *Conidiogenous cells* swollen towards the base, hyaline, smooth, tapering gradually towards the apex, which often forms a thin neck, monophialidic, (15–)17–21(–23) × 3–4 µm. *Conidia* aseptate, hyaline, smooth, arising from phialides at the apex of each neck, fusiform, (6–)6.5–8.5(–10) × 2–3 µm, with a mucous sheath.

Colonies on SDYA/4, slow-growing, attaining a diam. of 30 mm after 20 d at 25 °C. *Conidiogenous cells* swollen towards the base, hyaline, smooth, tapering gradually towards the apex, which often forms a thin neck, monophialidic or polyphialidic, (10–)12–15(–17) × (2–)2.5–3 µm. *Conidia* aseptate, hyaline, smooth, arising from phialides at the apex of each neck, fusiform, (7–)8.5–11.5(–13) × 2–3 µm, with a mucous sheath.

#### Distribution.

Thailand, only known from Khao Yai National Park.

#### Ecology.

Parasitic on a pair of termites from a reproductive caste (Order Isoptera: Family Termitidae, Subfamily Macrotermitinae) and these specimens were buried in the soil. The fungus emerged from the segment between the prothorax and mesothorax of one of the termite pairs.

#### Additional specimens examined.

THAILAND. Saraburi Province, Khao Yai National Park; 14°586'N, 100°998'E; on termite; 4 June 2017; S. Mongkolsamrit, U. Pinruan, P. Srikitikulchai, R. Promharn, S. Sommai (BBH45363, BBC86435).

#### Notes.

Four species, *O. asiatica*, *O. communis*, *O. pseudocommunis* and *O. pseudorhizoidea* look morphologically similar in having superficial perithecia and long wiry, pliant stroma emerging from the ground. In *O. asiatica* and *O. communis*, the stroma is dark brown, while in *O. pseudocommunis* and *O. pseudorhizoidea* it is cream to light brown. The perithecia in *O. communis*, *O. pseudocommunis* and *O. pseudorhizoidea* are larger than *O. asiatica*, but its ascospores are larger than in *O. pseudorhizoidea*.

### Ophiocordyceps brunneirubra

Taxon classificationFungiHypocrealesOphiocordycipitaceae

Tasanathai, Noisripoom, Luangsa-ard & Hywel Jones sp. nov.

ff07f067-1471-5e04-b5ba-6c78df2e12c0

MycoBank MB 831289

Figure 3

#### Typification.

THAILAND. Uthai Thani Province, Huai Kha Khaeng Wildlife Sanctuary; 15°605'N, 99°330'E; on termite; 28 August 2003; N.L. Hywel-Jones (holotype BBH 9008 dried culture; ex-type living culture: BCC14478). GenBank: ITS = MH754734, LSU = MH753688, *TEF* = GU797122, *RPB1* = MK751466, *RPB2* = MK214102

**Figure 3.**
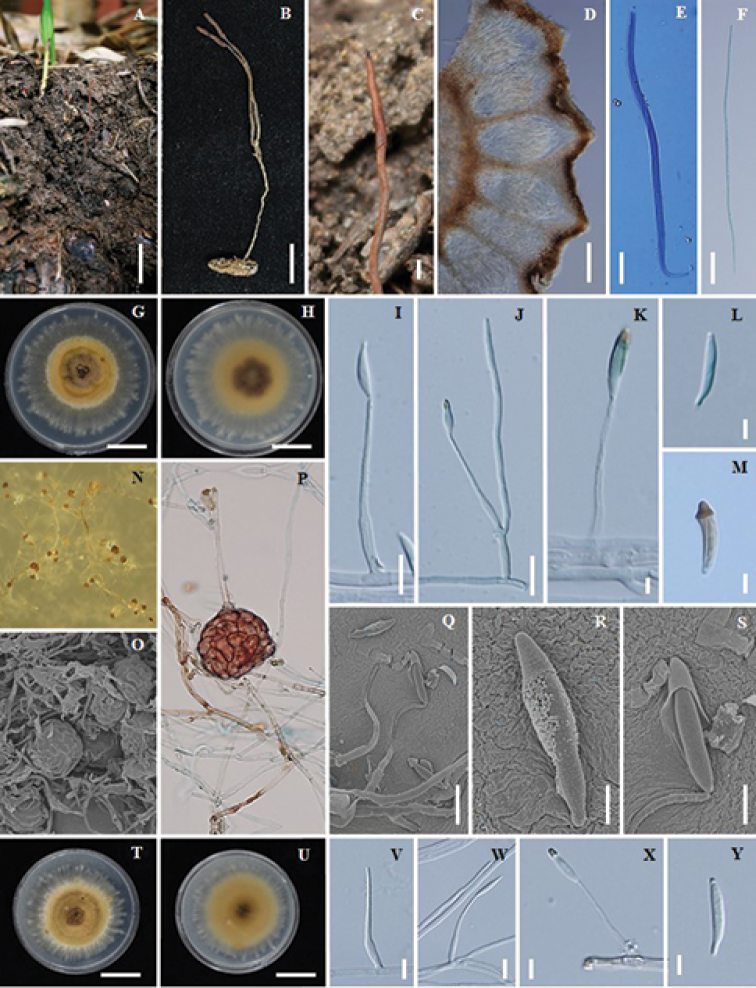
*Ophiocordyceps brunneirubra* (BBH 9008, BCC14478) **A, B** fungus on termite **C** part of stroma showing perithecia **D** immersed perithecia **E** asci **F** ascospore **G, H** colony on PDA at 20 d (**G**) colony obverse (**H**) colony reverse **I, J, K** phialides with conidia on PDA**L, M** conidia on PDA**N, P, O** sclerotia formed in culture **Q, R, S** scanning electron micrographs of phialides with conidia **T, U** colony on PSA at 20 d (**T**) colony obverse (**U**) colony reverse **V, W, X** phialides with conidia on PSA**Y** conidia on PSA. Scale bars: 25 mm (**A**); 15 mm (**B, G, H, T, U**); 1 mm (**C**); 130 μm (**D**); 10 μm (**I, Q, W**); 15 μm (**J**); 3 μm (**K, R**); 5 μm (**L**); 4 μm (**M, S, Y**); 6 μm (**V**); 7 μm (**X**).

#### Etymology.

‘*brunneirubra*’ referring to the reddish-brown appearance of the fertile head.

#### Description.

Stroma solitary, simple or branched, narrowly clavate, slender and wiry, up to 9.5 cm long, 0.5 mm wide. Fertile head cylindric, orange brown (oac642) to red brown (oac635), up to 8 mm long, 1 mm wide. *Perithecia* immersed, ovoid, ordinal in arrangement, (300–)334.5–400(–403) × (130–)138.5–178(–200) µm. *Asci* 8-spored, cylindrical, (155–)176–214.5(–225) × 4.5–7(–8) µm. *Ascospores* whole, filiform, 156.5–197.5 × 2–3 µm, with septa.

#### Culture characteristics.

Colonies on PDA, attaining a diam. of 25 mm within 20 d at 25 °C, orange (oac651) to orange brown (oac639). *Conidiogenous cells* monophialidic, arising from hyphae laterally or terminally, hyaline, tapering gradually or abruptly into a long slender neck, (32–)35.5–43.5(–50) µm long, (2–)2.5–3µm wide at the base, 1–1.5 µm wide at tip with warty surface. *Conidia* hyaline, one-celled, with a distinct gold cap covering the tip of the conidia, fusiform, (12–)13.5–15.5(–17) × 2–3 (–4) µm. Sclerotia formed in culture after 1 month, dark brown (oac635).

Colonies on PSA, attaining a diam. of 25 mm within 20 d at 25 °C, orange brown (oac716) to brown (oac721), reverse orange brown (oac721). *Conidiogenous cells* monophialidic, arising from hyphae laterally or terminally, hyaline, tapering gradually or abruptly into a long slender neck, (30–)32.5–39.5(–41) µm long, (2–)2.5–3.5(–4) µm wide at the base, 1–1.5 µm wide at tip with warty surface. *Conidia* hyaline, one-celled, arising from phialides, with a distinct gold cap covering the tip of the conidia, fusiform, (13–)14–16(–17) × 2–3 µm.

Colonies on SDYA/4, attaining a diam. of 25 mm within 20 d at 25 °C, dark brown (oac733), reverse orange brown (oac728). *Conidiogenous cells* monophialidic, arising from hyphae laterally or terminally, hyaline, tapering gradually or abruptly into a long slender neck, 25–40 µm long, 2–4 µm wide at the base, 1 µm wide at tip with warty surface. *Conidia* hyaline, one-celled, arising from phialides, with a distinct gold cap covering the tip of the conidia, fusiform, 12–15 × 2–3 µm.

#### Distribution.

Thailand, only known from Huai Kha Kaeng Wildlife Sanctuary.

#### Ecology.

Parasitic on a subterranean termite (Order Isoptera: Family Termitidae, Subfamily Macrotermitinae), collected from the soil. These termites belong to the reproductive caste (king or queen alates). The fungus emerged from between head and thoraxes of termite alates.

#### Additional specimens examined.

THAILAND. Uthai Thani Province, Huai Kha Khaeng Wildlife Sanctuary; at 15°605'N, 99°330'E; on termites; 28 Aug 2003; N.L. Hywel-Jones (BBH9009, BCC14477), (BBH9005, BCC14384).

#### Notes.

This species differs from other species on termites collected in Thailand in being singly infected by fungus instead of termite pairs and having immersed perithecia and red brown fertile terminal stroma. The species is not commonly found since it could easily be mistaken as a plant material sprouting from the ground. It is reminiscent of *O. brunneipunctata* but only on a different host. The shape of the conidia, like a banana with a hat or a cap, has never been seen in any kind of fungal spore morphology before.

### Ophiocordyceps khokpasiensis

Taxon classificationFungiHypocrealesOphiocordycipitaceae

Tasanathai, Noisripoom & Luangsa-ard sp. nov.

6da3ae70-6561-5eea-92fe-23fab9f162a5

MycoBank MB 831290

Figure 4

#### Typification.

THAILAND. Kalasin Province, Phu Si Than Wildlife Sanctuary, Khok Pa Si Community Forest; 16°562'N, 104°103'E; on termite; 14 June 2011; K. Tasanathai, P. Srikitikulchai, A. Khonsanit, K. Sansatchanon, W. Noisripoom (holotype BBH32173 dried culture; ex-type living culture: BCC48071). GenBank: ITS = MH754728, LSU = MH753682, *TEF* = MK284269, *RPB1* = MK214112

**Figure 4.**
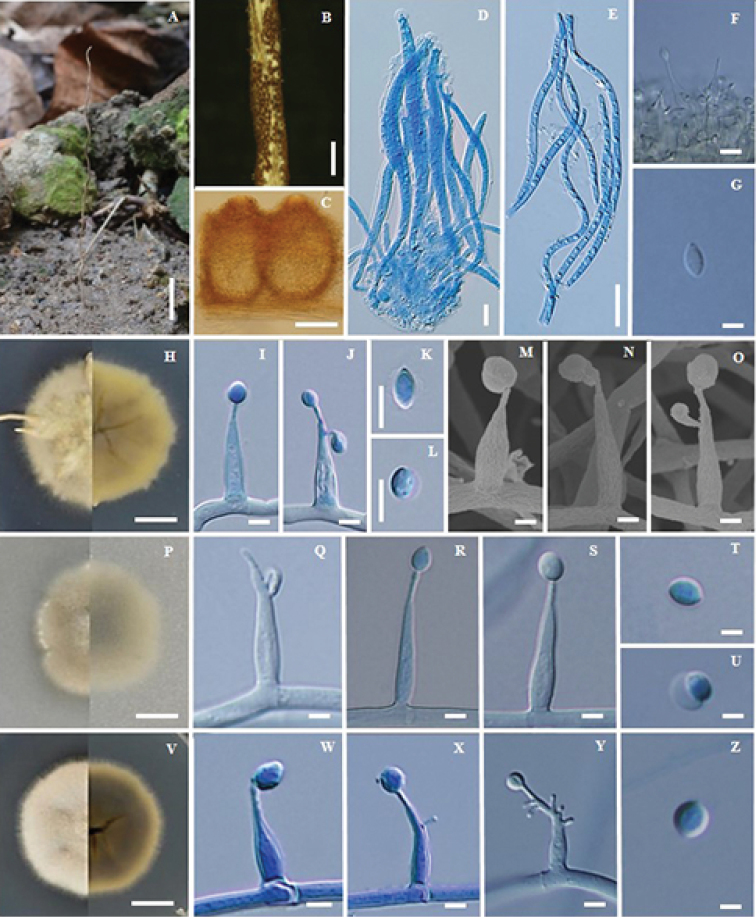
*Ophiocordyceps khokpasiensis* (BBH32173, BCC48071) **A** fungus on termite **B** part of stroma showing perithecia **C** pseudo-immersed perithecia **D** asci **E** ascospore **F** phialides with conidia from synnema **G** conidia from synnema **H** colony on PDA at 20 d colony obverse and reverse **I, J** phialides with conidia on PDA**K, L** conidium **M, N, O** scanning electron micrographs of phialides with conidia on PDA**P** colony on PSA at 20 d obverse and reverse **Q, R, S** phialides with conidia on PDA**T, U** conidium **V** colony on SDYA/4 at 20 d obverse and reverse **W, X, Y** phialides with conidia **Z** conidium. Scale bars: 2.5 cm (**A**); 1 mm (**B**); 100 µm (**C**); 5 µm (**D, G, I, J, K, L**); 20 µm (**E**); 6 µm (**F**); 7 mm (**H, P, V**); 3 µm (**M, N, O, Q, R, S, T, U**); 4 µm (**W, X, Y**); 2 µm (**Z**).

#### Etymology.

‘*khokpasiensis*’ referring to Khok Pa Si community forest, site of collection of type species.

#### Description.

Stroma solitary, simple, cylindrical, 16 cm long, 1 mm wide, brown (oac48-50), ca. 5.5 cm emerging above the leaf litter, ca. 10.5 cm buried in the soil. Asexual state (*Hirsutella*) produced ca. 1.5 cm at the terminal part of the stroma, light brown to grey. *Perithecia* pseudo-immersed, subglobose to broadly ellipsoidal, covering middle part of stroma, (200–)214–248.5(–250) × (120–)140–186(–200) µm. *Asci* 8-spored, filiform, (62.5–)86–115(–125) × 4–5 µm. *Ascospores* whole, filiform, (46–)51–74(–90) × 2–3 µm. Asexual state *Hirsutella*, phialides arising singly or laterally from the hyphae along the terminal part of the stroma, (8–)9–11(–12) × 3–4 µm. Conidia, hyaline, oval, 5–6.5(–7) × 2–3 µm.

#### Culture characteristics.

Colonies on PDA, attaining a diam. of 25.5 mm within 20 d at 25 °C, cream (oac900) to grey (oac893). *Conidiogenous cells* swollen towards the base, hyaline, smooth, tapering gradually towards the apex, which often forms a thin neck, monophialidic or polyphialidic, (15–)16.5–23(–28) × 3–4.5(–5) µm. *Conidia* arising from phialides at the apex of each neck, globose to oval, one-celled (4–)4.5–5.5(–6) × 2.5–4 µm, embedded in a mucous sheath.

Colonies on PSA, attaining a diam. of 24 mm within 20 d at 25 °C, white to grey (oac843). *Conidiogenous cells* swollen towards the base, hyaline, smooth, tapering gradually towards the apex, which often forms a thin neck, monophialidic or polyphialidic, (14–)15.5–22.5(–28) × 3–4.5(–5) µm. *Conidia* arising from phialides at the apex of each neck, globose to oval, one-celled 4–5(–6) × (2–)2.5–3.5(–5) µm, embedded in a mucous sheath.

Colonies on SDYA/4, attaining a diam. of 25 mm within 20 d at 25 °C, grey to brown (oac473). *Conidiogenous cells* swollen towards the base, hyaline, smooth, tapering gradually towards the apex, which often forms a thin neck, monophialidic or polyphialidic, (9–)11.5–15.5(–19) × (2–)3–3.5(–4) µm. *Conidia* arising from phialides at the apex of each neck, globose to oval, one celled 3.5–4.5(–5) × 2.5–3 (–3.5) µm, embedded in a mucous sheath.

#### Distribution.

North-eastern Thailand.

#### Ecology.

Parasitic on a pair of termites from a reproductive caste (Order Isoptera: Family Termitidae, Subfamily Macrotermitinae) and these specimens were buried in the soil. The fungus emerged from the segment between the prothorax and mesothorax of one of the termite pairs.

#### Additional specimens examined.

THAILAND. Saraburi Province, Namtok Samlan National Park (Phra Buddha Chai); 14°526'N, 100°9'E; on termite; 15 June 1996; Hywel-Jones, NL (BBH5116, BCC1764). Kalasin Province: Phu Si Than Wildlife Sanctuary, Khok Pa Si Community Forest; 16°562'N, 104°103'E; on termite; 14 June 2011; K. Tasanathai, P. Srikitikulchai, A. Khonsanit, K. Sansatchanon, W. Noisripoom (BBH32173, BCC48072).

#### Notes.

Other *Ophiocordyceps* species reported on termites with pseudo-immersed perithecia are *O. mosingtoensis* and *O. termiticola*. *O. khokpasiensis* and *O. termiticola* shares similarity in the colour of the perithecia but in *O. termiticola*, the perithecia are denser while it is loosely arranged in *O. khokpasiensis*. *O. mosingtoensis* produces a more robust stroma compared to *O. khokpasiensis* and *O. termiticola*. The gross morphology of *O. khokpasiensis* is similar to *O. asiatica*, *O. communis*, *O. pseudocommunis* and *O. pseudorhizoidea*. However, all these other species produce superficial perithecia.

### Ophiocordyceps mosingtoensis

Taxon classificationFungiHypocrealesOphiocordycipitaceae

Tasanathai, Noisripoom & Luangsa-ard sp. nov.

9fc2e9d4-9ae3-5f64-9f42-351bc8647514

MycoBank MB 831291

Figure 5

#### Typification.

THAILAND. Nakhon Ratchasima Province, Khao Yai National Park; 14°711'N, 101°421'E; on termite; 17 June 2009; K. Tasanathai, P. Srikitikulchai, S. Mongkolsamrit, T. Chohmee, R. Ridkaew, N.L. Hywel-Jones (holotype BBH26809 dried culture; ex-type living culture, BCC36921). GenBank: ITS = MH754731, LSU = MH753685, *TEF* = MK284272, *RPB1* = MK214116, *RPB2* = MK214099

**Figure 5.**
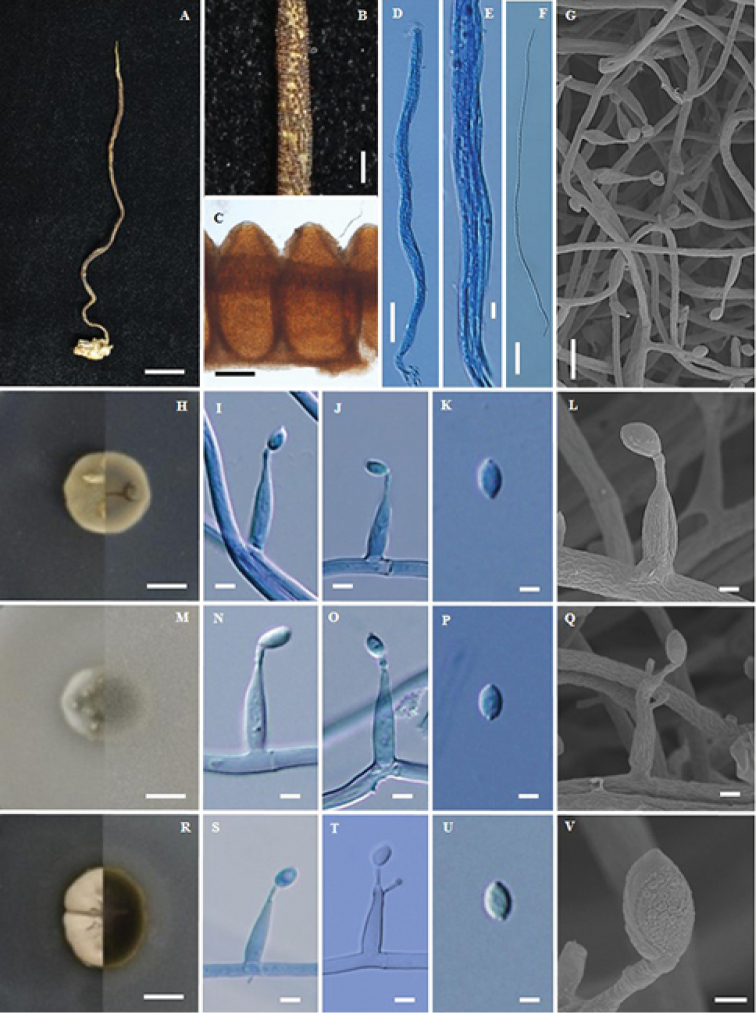
*Ophiocordyceps mosingtoensis* (BBH26809, BCC36921) **A** stroma of fungus emerging from termite **B** part of stroma showing perithecia **C** pseudo-immersed perithecia **D, E** ascus **F** ascospore **G, L, Q, V** scanning electron micrographs of phialides with conidia on PDA**H** colony on PDA at 20 d obverse and reverse **I, J** phialides with conidia **K** conidium **M** colony on PSA at 20 d obverse and reverse **N, O** phialides with conidia **P** conidium **R** colony on SDYA/4 at 20 d obverse and reverse **S, T** phialides with conidia **U** conidium. Scale bars: 10 mm (**A**); 1 mm (**B**); 150 µm (**C**); 25 µm (**D**); 4 µm (**E**); 30 µm (**F**); 10 µm (**G**); 8 mm (**H, M, R**); 3 µm (**I, J, N, O, S, T**); 2 µm (**K, L, P, Q, U**); 1 µm (**V**).

#### Etymology.

‘*mosingtoensis*’ referring to name after the type locality.

#### Description.

Stroma solitary, simple, cylindrical, up to 11 cm long, 1 mm wide, brown (oac 48-50), ca. 8.5 cm emerging above the leaf litter, ca. 2.5 cm buried in the soil. Asexual state (*Hirsutella*) produced ca. 1 cm at the terminal part of the stroma, light brown to grey. *Perithecia* pseudo-immersed, broadly ovoid covering middle part of stroma, (400–)414–469 (–500) × (200–)208–263(–300) µm. *Asci* 8-spored, filiform, (187.5–) 217–265(–287.5) × 4.5–6.5(–7.5) µm with cap, 2 µm. *Ascospores* whole, filiform, (230–)240–291(–315) × 1.5–3 µm, with septa.

#### Culture characteristics.

Colonies on PDA, attaining a diam. of 16 mm within 20 d at 25 °C, cream (oac872) to grey (oac909). *Conidiogenous cells* swollen towards the base, hyaline, smooth, tapering gradually towards the apex, which often forms a thin neck, monophialidic, (10–)12.5–16 (–17) × (2–) 2.5–3 µm. *Conidia* arising from phialides at the apex of each neck, oval, 3–4.5(–5) × 2–2.5(–3) µm.

Colonies on PSA, attaining a diam. of 17 mm within 20 d at 25 °C, white to grey (oac872). *Conidiogenous cells* swollen towards the base, hyaline, smooth, tapering gradually towards the apex, which often forms a thin neck, monophialidic, (10–)11.5–15(–17) × (2–)2.5–3.5(–4) µm. *Conidia* arising from phialides at the apex of each neck, oval, (3–)3.5–5(–5.5) × 2–3 µm.

Colonies on SDYA/4, attaining a diam. of 17 mm within 20 d at 25 °C, white to grey (oac802). *Conidiogenous cells* swollen towards the base, hyaline, smooth, tapering gradually towards the apex, which often forms a thin neck, monophialidic or polyphialidic, (9–)10.5–14.5(–17) × (2–)2.5–3 µm. *Conidia* arising from phialides at the apex of each neck, oval, (3–)3.5–4.5(–5) × 2–3 µm.

#### Distribution.

Thailand, only known from Khao Yai National Park.

#### Ecology.

Parasitic on a pair of termites from a reproductive caste (Order Isoptera: Family Termitidae, Subfamily Macrotermitinae) and these specimens were buried in the soil. The fungus emerged from the segment between the prothorax and mesothorax of one of the termite pairs.

#### Additional specimens examined.

THAILAND. Nakhon Ratchasima Province, Khao Yai National Park; 14°711'N, 101°421'E; on termite; 18 June 2008; J.J. Luangsa-ard, K. Tasanathai, S. Mongkolsamrit, B. Thongnuch, P. Srikitikulchai, R. Ridkaew (BBH 23860, BCC 30904).

#### Note.

*O. mosingtoensis* has a sturdier, robust stroma compared with *O. termiticola* and *O. khokpasiensis* which also produce pseudo-immersed perithecia.

### Ophiocordyceps pseudocommunis

Taxon classificationFungiHypocrealesOphiocordycipitaceae

Tasanathai, Noisripoom & Luangsa-ard sp. nov.

70bc8020-b538-5050-98de-deb4e25f77f6

MycoBank MB 831351

Figure 6

#### Typification.

THAILAND. Nakhon Nayok Province, Khao Yai National Park; 14°163'N, 101°268'E; on termite; 13 July 2004; S. Sivichai, K. Tasanathai, N. Boonyuen, P. Puyngain (holotype BBH10001 dried culture; ex-type living culture, BCC16757). GenBank: ITS = MH754733, LSU = MH753687, *TEF* = MK284274, *RPB1* = MK214117, *RPB2* = MK214101

**Figure 6.**
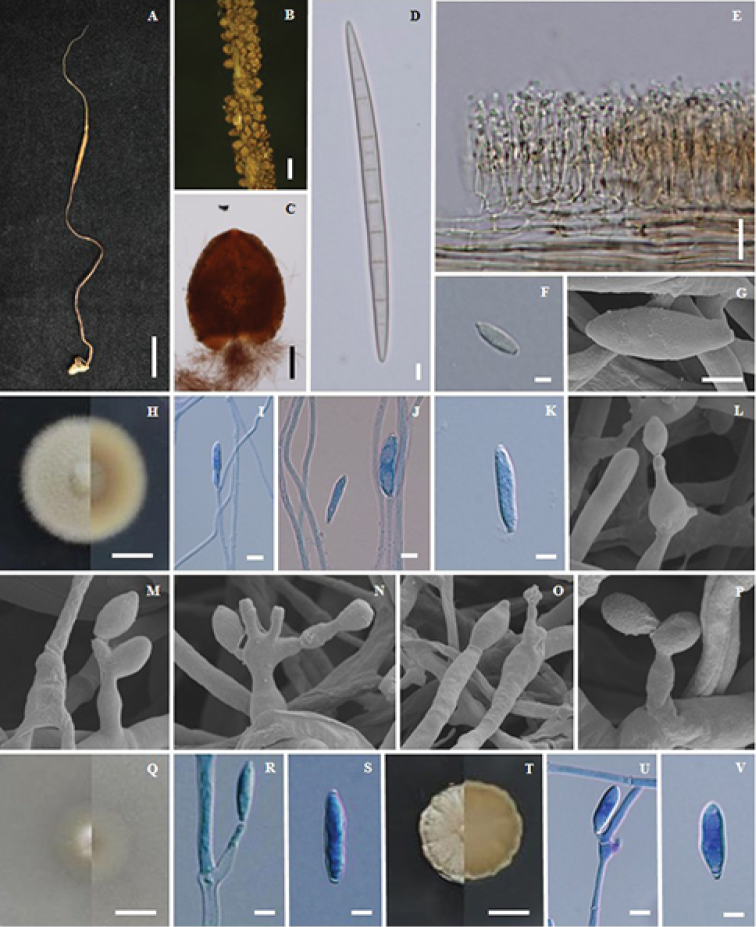
*Ophiocordyceps pseudocommunis* (BBH10001, BCC16757) **A** stroma of fungus emerging from termite **B** part of stroma showing superficial perithecia **C** perithecium **D** ascospore **E** phialides with conidia from synnema **F** conidia from synnema **G, L, M, N, O, P** scanning electron micrographs of phialides with conidia on PDA**H** colony on PDA at 20 d obverse and reverse **I, J** phialides with conidia on PDA**K** conidium **Q** colony on PDA at 20 d obverse and reverse **R** phialides with conidia on PSA**S** conidium **T** colony on SDYA/4 at 20 d obverse and reverse **U** phialides with conidia **V** conidium. Scale bars: 10 mm (**A**); 0.5 mm (**B**); 150 µm(**C**); 6 µm (**D**); 7 µm (**E**); 2 µm (**F**); 4 µm (**G**); 8 mm (**H, Q, T**); 8 µm (**I**); 5 µm (**J, K, U, V**); 3 µm (**R, S**).

#### Etymology.

‘*pseudocommunis*’ referring to close affinity to *Ophiocordyceps communis*.

#### Description.

Stroma solitary, simple, cylindrical, 21.5 cm long, 0.5 mm wide, brown (oac48-50), ca. 12 cm emerging above the leaf litter, ca. 9 cm buried in the soil. Asexual state (Hymenostilbe-like) produced ca. 5 cm at the terminal part of the stroma, light brown to brown. *Perithecia* superficial, subglobose, covering middle part of the stroma, (520–)536.5–596.5(–600) × (360–)373.5–425 (–440) µm. *Asci*, 8-spored, filiform, 160–164.5(–165) × 14–17 µm. *Ascospores* whole, filiform, (107.5–)120.5–138 (–147.5) × (6–)6.5–7 (7.5) µm, with 7–8 septa. Asexual state Hymenostilbe-like, conidiogenous cells forming a compact hymenium-like layer and had two to four denticles at their apices, cylindrical to clavate, (17–)18.5–21(–22) × (2–)2.5–7.5(–8) µm. Conidia, hyaline, fusiform, (6–)6.5–7.5(–8) × 2–3 µm.

#### Culture characteristics.

Colonies on PDA, attaining a diam. of 26.5 mm within 20 d at 25 °C, white (oac909) to grey (oac851). *Conidiogenous cells* arising from hyphae laterally or terminally, hyaline, tapering gradually or abruptly into a long slender neck. *Conidia* hyaline, septate (2–3), arising from phialides at the apex of each neck, fusiform, (13–)14.5–20.5(–27) × (3–)3.5–5 µm.

Colonies on PSA, attaining a diam. of 15 mm within 20 d at 25 °C, white (oac909) to grey (oac851). *Conidiogenous cells* arising from hyphae laterally or terminally, hyaline, tapering gradually or abruptly into a long slender neck. *Conidia* hyaline, septate (1–4), arising from phialides at the apex of each neck, fusiform, (7–)9–15.5(–20) × (2–)2.5–4 µm.

Colonies on SDYA/4, attaining a diam. of 19 mm within 20 d at 25 °C, cream (oac816) to brown (oac781). *Conidiogenous cells* arising from hyphae laterally or terminally, hyaline, tapering gradually or abruptly into a long slender neck. *Conidia* hyaline, septate, arising from phialides at the apex of each neck, fusiform, (7–)9–18.5(–27) × (3–)3.5–6(–8) µm.

#### Distribution.

Only reported from Khao Yai National Park.

#### Ecology.

Parasitic on a pair of termites from a reproductive caste (Order Isoptera: Family Termitidae, Subfamily Macrotermitinae) and these specimens were buried in the soil. The fungus emerged from the segment between the prothorax and mesothorax of one of the termite pairs.

#### Additional specimens examined.

THAILAND. Nakhon Ratchasima Province, Khao Yai National Park; 14°711'N, 101°421'E; on termite; 22 July 2003; R. Nasit, N.L. Hywel-Jones, J.W. Spatafora (NHJ12581, NHJ12582).

### Ophiocordyceps pseudorhizoidea

Taxon classificationFungiHypocrealesOphiocordycipitaceae

Tasanathai, Noisripoom & Luangsa-ard sp. nov.

89c50ae7-61e5-5efe-9a8c-1297b5b5e27e

MycoBank MB 830982

Figure 7

#### Typification.

THAILAND. Khonkaen Province, Phu Wiang National Park; 16°799'N, 102°279'E; on termite; 17 July 2017; K. Tasanathai, S. Mongkolsamrit, W. Noisripoom (holotype BBH45361 dried culture; ex-type living culture, BCC86431). GenBank: ITS = MH754721, LSU = MH753674, *TEF* = MK284262, *RPB1* = MK751469, *RPB2* = MK214090

**Figure 7.**
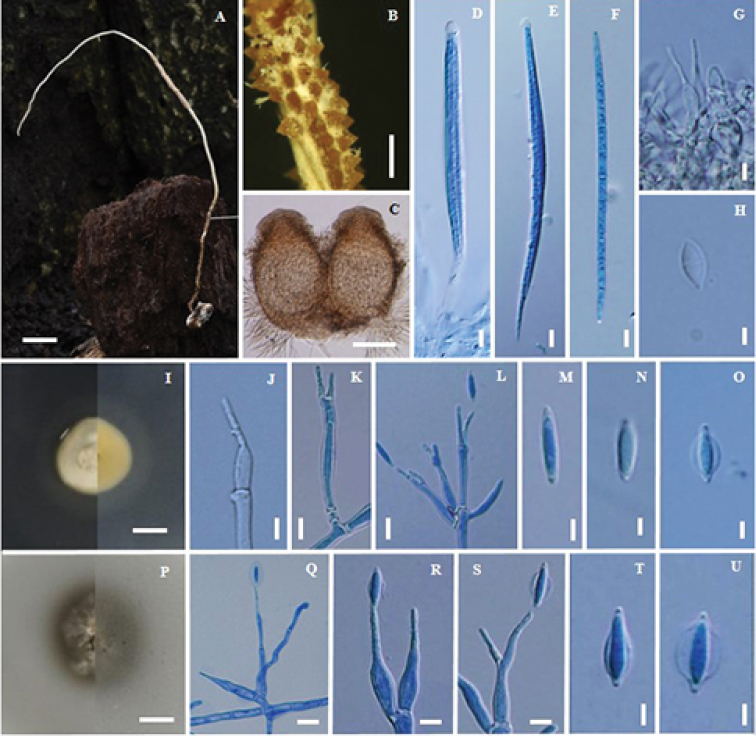
*Ophiocordyceps pseudorhizoidea* (BBH45361, BCC86431) **A** stroma of fungus emerging from termite **B** part of stroma showing perithecia **C** perithecia **D, E** ascus **F** ascospore **G, H** phialides with conidia from synnema **I** colony on PDA at 20 d obverse and reverse **J, K, L** phialides with conidia on PDA**M, N, O** conidium **P** colony on PSA at 20 d obverse and reverse **Q, R, S** phialides with conidia on PSA**T, U** conidia with mucous sheath. Scale bars: 15 mm (**A**); 1 mm (**B**); 120 μm (**C**); 8 μm (**D, E**); 10 μm (**F, G**); 3 μm (**H, R**); 6 mm (**I, P**); 5 μm (**J, K, L, Q**); 2 μm (**M, N, O, T, U**); 4 μm (**S**).

#### Etymology.

‘*pseudorhizoidea*’ referring to close affinity to what was called *Ophiocordyceps rhizoidea* on termites by NHJ.

#### Description.

Stroma solitary, simple, filiform, up to 21 cm long, 1 mm wide, light-brown (oac675), ca. 15 cm emerging above leaf litter, 5.5 cm buried in the soil. Asexual state (*Hirsutella*) produced at the terminal part of the stroma, ca. 6 cm long, light brown to grey. *Perithecia* superficial, ovoid, covering the middle part of stroma, (280–) 287.5–315.5 (–390) × (160–) 177–209.5 (–220) µm. *Asci* 8-spored, cylindrical, 120–150 × 5–7 µm with cap, 3–4 × 4–5 µm. *Ascospores* whole, filiform, (65–) 69.5–78.5 (–82.5) × 2–2.8 (–3) µm, with septate. Asexual state *Hirsutella*. Phialides (10–)15.5–23.5(–26) × 3–4(–5) µm, conidia hyaline, fusiform, (5–)5.5–6.5(–7) × 3–4 µm.

#### Culture characteristics.

Colonies on PDA, attaining a diam. of 10 mm within 20 d at 25 °C, cream to grey (oac844), reverse oac772 to oac815. *Conidiogenous cells* monophialidic, arising from hyphae laterally or terminally, hyaline, tapering gradually or abruptly into a long slender neck, (9–)10.5–17.5(–21) µm long, 2–3.2(–4) µm wide at the base, 1–1.5 µm wide at tip with warty surface. *Conidia* hyaline, one-celled, fusiform, (5–)6.5–8.5(–10) × 1–2 µm. with mucous sheath.

Colonies on PSA, attaining a diam. of 10 mm within 20 d at 25 °C, (oac841) to (oac843), reverse (oac868). *Conidiogenous cells* monophialidic cells arising from hyphae laterally or terminally, hyaline, tapering gradually or abruptly into a long slender neck, (10–)12–16.5(–19) µm long, 2–3 µm wide at the base, 1–1.5 µm wide at tip with warty surface. *Conidia* hyaline, one-celled, arising from phialides, fusiform, (6–)6.5–8(–8.5) × 1.5–2.5(–3) µm with mucous sheath.

Colonies on SDYA/4, attaining a diam. of 10 mm within 20 d at 25 °C, oac844, reverse oac722 in middle to oac815. *Conidiogenous cells* monophialidic cells arising from hyphae laterally or terminally, hyaline, tapering gradually or abruptly into a long slender neck, (13–)17–25.5(–30) µm long, (3–)3.5–4 µm wide at the base, 1 µm wide at tip with warty surface. *Conidia* hyaline, one-celled, arising from phialides, fusiform, (6–)7.5–9(–10) × 1–2 µm with mucous sheath.

#### Distribution.

Thailand.

#### Ecology.

Parasitic on a pair of termites from a reproductive caste (Order Isoptera: Family Termitidae, Subfamily Macrotermitinae) and these specimens were buried in the soil. The fungus emerged from the segment between the prothorax and mesothorax of one of the termite pairs.

#### Additional specimens examined.

THAILAND. Chanthaburi Province, Khao Soi Dao Wildlife Sanctuary; 13°136'N, 102°218'E; on termite; 8 June 2011; K. Tasanathai, P. Srikitikulchai, S. Mongkolsamrit, A. Khonsanit, K. Sansatchanon (BBH31259, BCC 48879).

#### Notes.

Like *O. communis* and *O. pseudocommunis*, this species shows similarity to *O. rhizoidea*. However, von Hohnel’s description of the host in *O. rhizoidea* was a Coleoptera larva. *O. rhizoidea* has longer and wider asci and ascospores than *O. pseudorhizoidea*, while in *O. communis* and *O. pseudocommunis*, they are distinctly longer (Table 2).

**Table 2.** Morphological comparisons of closely related *Ophiocordyceps* species used in this study

Species	Host	Stromata (cm)	Perithecia (µm)	Asci (µm)	Ascospores (µm)	Reference
* Ophiocordyceps asiatica *	Termites	solitary, simple, filiform, up to 15 long orange brown	superficial, globose to subglobose 240–320 × 180–260	filiform 92.5–175 × 5–6.3	whole with septate 90–132.5 × 1–2	This study
* Ophiocordyceps brunneirubra *	Termites	solitary, simple or branched, narrowly clavate, slender and wiry, 9.5 cm long, orange brown to red brown	Immersed, ovoid, 300–400 × 130–200	cylindrical, 155–225 × 4.5–8	filiform, whole with septate, 156.5–197.5 × 2–3	This study
* Ophiocordyceps khokpasiensis *	Termites	solitary, simple cylindrical, 16 cm long, brown	pseudo-immersed, subglobose 200–250 × 120–200	filiform, 62.5–125 × 4–5	filiform, whole, 46–90 × 2–3	This study
* Ophiocordyceps mosingtoensis *	Termites	solitary simple cylindrical, 11 cm long, brown to grey	pseudo-immersed, ovoid 400–500 × 200–300	filiform, 187.5–287.5 × 4.5–7.5	whole with septate, 230–315 × 1.5–3	This study
* Ophiocordyceps pseudocommunis *	Termites	solitary simple cylindrical , 21 cm long, brown	superficial, subglobose 520–600 × 360–440	filiform, 160–165 × 14–17	whole with 7–8 septa, 107.5–147.5 × 6–7.5	This study
* Ophiocordyceps communis *	Termites	solitary simple filiform, 5-13 cm long, yellow brown	superficial 285-675 × 195-390	filiform, 215-250 × 15	filiform, whole, 100–180 × 5–6	Sung et al. 2007
* Ophiocordyceps pseudorhizoidea *	Termites	solitary, simple, filiform, up to 21 cm long, light brown	superficial, ovoid 280–390 × 160–220	cylindrical, 120–150 × 5–7	whole with septate 65–82.5 × 2–3	This study
* Ophiocordyceps rhizoidea *	Coleoptera larva	simple, solitary, 7–8 cm long, 0.5-1 mm	superficial 360 × 300	160-210 × 13-16	ca 80 × 5-7	von Höhnel, 1909
* Ophiocordyceps termiticola *	Termites	solitary, simple, filiform, up to 14 cm long yellow brown	pseudo-immersed, globose to subglobose 200–280 × 150–250	filiform 62.5–110 × 4–6	filiform, whole, 85 × 2	This study

### Ophiocordyceps termiticola

Taxon classificationFungiHypocrealesOphiocordycipitaceae

Tasanathai, Noisripoom & Luangsa-ard sp. nov.

ca947b09-558d-514f-aaa7-7aeba2962c64

MycoBank MB 831296

Figure 8

#### Typification.

THAILAND. Kanchanaburi Province, Khao Laem National Park; 14°746'N, 98°625'E; on termite; 20 June 1995; N.L. Hywel-Jones, R. Nasit, S. Sivichai (holotype BBH5634 dried culture; ex-type living culture, BCC 1920). GenBank: ITS = MH754724, LSU = MH753678, *TEF* = MK284265, *RPB1* = MK214108, *RPB2* = MK214094

**Figure 8.**
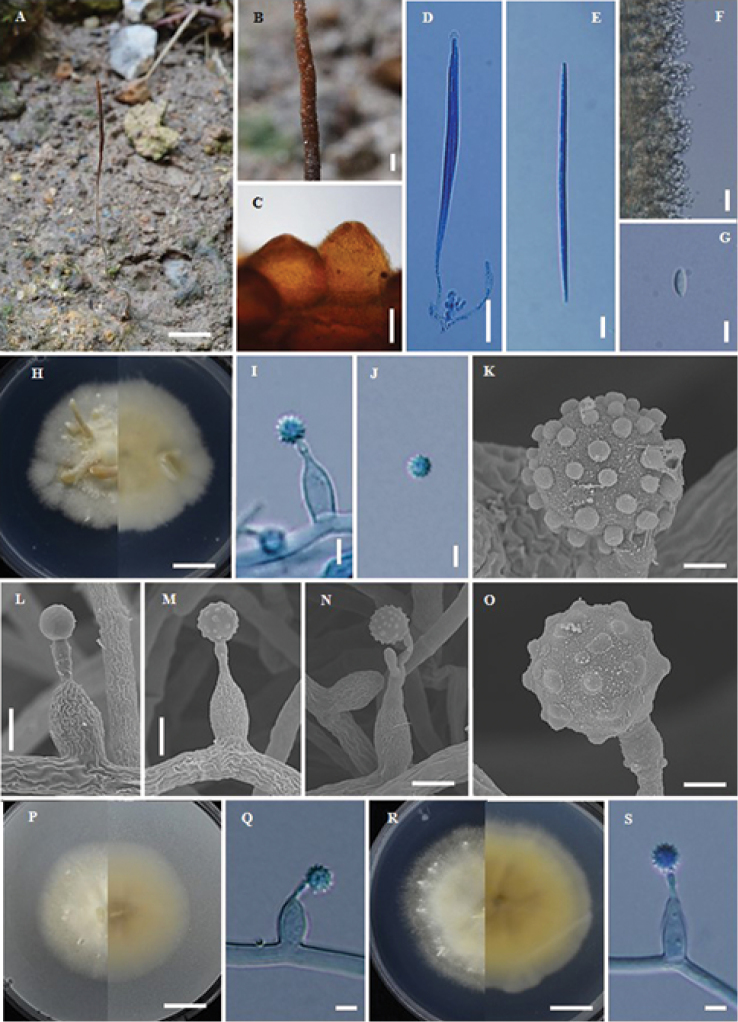
*Ophiocordyceps termiticola* (BBH5634, BCC 1920) **A** stroma of fungus emerging from termite **B** part of stroma showing perithecia **C** perithecia **D** ascus **E** ascospore **F** phialides with conidia on synnema **G** conidium **H** colony on PDA at 20 d obverse and reverse **I** phialides with conidia on PDA**J** conidium **K–O** scanning electron micrographs of phialides with conidia on PDA**P** colony on PSA at 20 d obverse and reverse **Q** phialides with conidia on PSA**R** colony on SDYA/4 at 20 d obverse and reverse **S** phialides with conidia. Scale bars: 2 cm (**A**); 1 μm (**B, K, O**); 100 μm (**C**); 15 μm (**D**); 8 μm (**E**); 5 μm (**F, G**); 7 mm (**H, P, R**); 3 μm (**I, J, L, M, N, Q, S**).

#### Etymology.

‘*termiticola*’ referring to the host family, Termitidae.

#### Description.

Stroma solitary, simple, filiform, up to 14 cm long, 1 mm wide, yellow-brown, ca. 6 cm emerging above the leaf litter, ca. 8 cm buried in the soil. Asexual state (Hymenostilbe-like) produced ca. 1 cm at the terminal part of the stroma, grey. *Perithecia* pseudo-immersed, globose to subglobose, produced on one-third of the terminal part of the stroma ending near the apex, (200–)225–261(–280) × (150–)178–229(–250) µm. *Asci* 8-spored, filiform, (62.5–)76.5–100.5(–110) × (4–)4.5–5.5(–6) µm. *Ascospores* whole, filiform, 85 × 2 µm, Asexual state Hymenostilbe-like, conidiogenous cells formed a compact hymenium-like layer and had from two to four denticles at their apices, cylindrical to clavate, (10–)11.5–16(–17) × 3–5(–6) µm. Conidia, hyaline, fusiform 7 × 3 µm.

#### Culture characteristics.

Colonies on PDA, attaining a diam. of 28 mm within 20 d at 25 °C, grey (oac781) to pale grey (oac851). *Conidiogenous cells* monophialidic to polyphialidic, arising from hyphae laterally, with an inflated base (7–)7.5–10(–11) × (2.5–) 3–3.5(–4) µm. *Conidia* hyaline, globose, 2.5–3 (–3.5) µm, one-celled with warty surface.

Colonies on PSA, attaining a diam. of 22 mm within 20 d at 25 °C, white to pale grey, cotton-like. *Conidiogenous cells* monophialidic to polyphialidic, hyaline, smooth, with an inflated base (7–)8–10.5(–13) × 3–4 (–5) µm. *Conidia* hyaline, globose, (2–)2.7–3.4(–4) µm, one celled with warty surface.

Colonies on SDYA/4, attaining a diam. of 29 mm within 20 d at 25 °C, grey to pale grey (oac851). *Conidiogenous cells* monophialidic to polyphialidic, hyaline, smooth, with an inflated base (7–)8–10.5(–13) × 3–4 µm. *Conidia* hyaline, globose, 3–3.5(–4) µm, one-celled with warty surface.

#### Distribution.

Thailand.

#### Ecology.

Parasitic on a pair of termites from a reproductive caste (Order Isoptera: Family Termitidae, Subfamily Macrotermitinae) and these specimens were buried in the soil. The fungus emerged from the segment between the prothorax and mesothorax of one of the termite pairs.

#### Additional specimens examined.

THAILAND. Chanthaburi Province, Khao Soi Dao Wildlife Reserve; 13°136'N, 102°218'E; on termite; 20 June 1996; R. Nasit, S. Sivichai, K. Tasanathai (BBH5179, BCC1770).

#### Notes.

Both *O. termiticola* and *O. khokpasiensis* produce pseudo-immersed reddish perithecia on a stroma. In *O. termiticola*, the perithecia are tightly packed, while in *O. khokpasiensis*, they are loosely aggregated and the length of the anamorphic layer at the end of the fertile part is longer in the latter.

## Discussion

Out of the 230+ species of *Ophiocordyceps* worldwide, less than 10 species occur on termites. The majority of these species produce cylindrical, wiry to pliant, mostly simple, seldom multiple, stromata. Species found in Africa and Mexico, *O. bispora* (*Cordycepioideus bisporus*) and *O. octospora* (*Cordycepioideus octosporus*), produce thick-walled, multiseptate ascospores, suggesting an adaptation to the harsh environmental conditions in these countries (Ochiel et al. 1997; Blackwell and Gilbertson 1981, 1984). All termite pathogenic species in Thailand including *O. asiatica*, *O. brunneirubra*, *O. communis*, *O. khokpasiensis*, *O. mosingtoensis*, *O. pseudocommunis*, *O. pseudorhizoidea* and *O. termiticola* produce filiform, multiseptate, whole ascospores on predominantly superficial and pseudo-immersed perithecia. The dark to pallidly coloured stroma of these species are cylindrical, wiry and pliant and the anamorph is produced at the terminal part of the stroma, after the fertile part.

Interestingly, our results clearly present *Ophiocordyceps* species occurring on reproductive castes of termites, especially subterranean termite species in the Family Termitidae, Subfamily Macrotermitinae. All species of subterranean termites construct their nests below ground or build mounds above ground and excavate their foraging tunnel in several ways (Eggleton 2010; Ahmad et al. 2018). Usually, the reproductive caste of termites, i.e. flying termites, includes male and female swarms during mating season at the start of the rainy season. The winged queen emerges from the colony for her nuptial flight or the mating flight, releasing pheromones to attract the males to mate. When the male finds the queen, they do a tandem run that lasts for as long as the pair finds a suitable place to start a new colony, during which they shed their wings. In termites, both male and female are the same size (Howard and Thorne 2010; Ahmad et al. 2018). Specimens of termites might have been infected by *Ophiocordyceps* species after their nuptial flight, when they bury themselves in the ground to establish a nesting area for starting a new colony.

Fungi represent a silent threat to the termite community. Termites have many predators, such as other amphibians (toads), birds, reptiles (lizards, gecko, snakes), small mammals, rodents and even humans. The percentage of the infection to these reproductive castes may be low in comparison to the individuals in a termite swarm, however, only few survive or evade the imminent threat of arthropods and other animals. Eventually, the number of infections caused by *Ophiocordyceps* becomes significant when only a few can actually survive to start a new colony.

The number of available morphological characters needed to delimit species in fungi are so limited and this may be an important reason why cryptic species are abundant in Kingdom Fungi, i.e. morphologically indistinguishable biological/phylogenetic units present within taxonomic species (Balasundaram et al. 2015) or, as Bickford et al. (2007) put it: ‘two or more distinct species that are erroneously classified (and hidden) under one species name’. Many species of entomopathogenic fungi in Ophiocordycipitceae belong to species complexes or are cryptic species. Zombie ant pathogens in *Ophiocordyceps* have all been classified as *Ophiocordyceps unilateralissensu lato* until morphological and molecular studies, including host identification, were completed (Araujo et al. 2015, 2018; Luangsa-ard et al. 2010; Kobmoo et al. 2012, 2015). The use of DNA-based molecular analyses has subsequently uncovered several new species in the genus (Khonsanit et al. 2018; Luangsa-ard et al. 2018). In culture, the conidiogenous cells of these termite pathogens produce phialides that are either monophialidic or have several lateral necks. The anamorphs of these species do not always form *Hirsutella* asexual states but more of an intermediate between *Hirsutella* and *Hymenostilbe*. This could either be a transition into a different genus or forming a diverging lineage in *Ophiocordyceps* – in the process of a speciation event or that the production of these anamorphs are so plastic that they cannot be used in taxonomy.

The knowledge that *Ophiocordyceps* species infect reproductive castes of termites can be used as basic information to study the biological control of subterranean termite pests and to better implement them. All specimens of termites collected are subterranean termites and produce relatively fast growing synnemata with numerous infectious propagules (ascopores) which can be developed further for biological control strategies.

## Supplementary Material

XML Treatment for Ophiocordyceps asiatica

XML Treatment for Ophiocordyceps brunneirubra

XML Treatment for Ophiocordyceps khokpasiensis

XML Treatment for Ophiocordyceps mosingtoensis

XML Treatment for Ophiocordyceps pseudocommunis

XML Treatment for Ophiocordyceps pseudorhizoidea

XML Treatment for Ophiocordyceps termiticola
